# Metformin and cancer: *Quo vadis et cui bono?*

**DOI:** 10.18632/oncotarget.10262

**Published:** 2016-06-23

**Authors:** Javier A. Menendez, Begoña Martin-Castillo, Jorge Joven

**Affiliations:** ^1^ Metabolism and Cancer Group, ProCURE (Program Against Cancer Therapeutic Resistance), Catalan Institute of Oncology, Girona, Catalonia, Spain; ^2^ Molecular Oncology Group, Girona Biomedical Research Institute (IDIBGI), Girona, Catalonia, Spain; ^3^ Unit of Clinical Research, Catalan Institute of Oncology, Girona, Catalonia, Spain; ^4^ Unitat de Recerca Biomèdica, Hospital Universitari de Sant Joan, IISPV, Universitat Rovira i Virgili, Campus of International Excellence Southern Catalonia, Reus, Spain

**Keywords:** metformin, cancer, metabolism, pharmacokinetics

## Abstract

How many lives have already been saved by the anti-cancer drug metformin? Inadvertently perhaps, among the millions of type 2 diabetics with occult or known cancers and who have been prescribed metformin since the 1950s, thousands may have benefited from the anticancer properties of this first-line pharmacotherapy. *Quo vadis?* Now, researchers aim to move metformin from a non-targeted stage of cancer therapy that has been mostly developed retrospectively and empirically into a targeted therapy by following a biological rationale and a predefined mechanism of action. But, who might benefit from metformin? *Cui bono?* Because metformin is on the leading edge of a new generation of cancer metabolism-targeted therapies, perhaps it is the right time to provide solutions to the challenges that metformin and other onco-biguanides will face in the coming years before becoming incorporated into the therapeutic armamentarium against cancer.

How many lives have already been saved by the anti-cancer drug metformin?

In a highly prevalent disease such as breast cancer (BC), a moderate improvement in survival rates would be enough to save thousands of lives worldwide if a given therapeutic approach is widely available and is accompanied by a benefit to a significant proportion of women developing the disease. When considering insulin as one of the most plausible biological mechanisms underlying the link between obesity and diabetes and a significantly increased risk of BC mortality, it is remarkable that a lowering of insulin by 25% is associated with a 5% absolute improvement in BC mortality [1]. Given that metformin can reduce fasting insulin levels 10-30% in women with early BC [2], one could speculate that metformin may have saved thousands of lives among the millions of diabetic women receiving metformin in the last 60 years. To put these putative numbers into a relevant scenario, we should acknowledge that a shift in the treatment paradigm of adjuvant tamoxifen treatment from the more traditional 5 years to 10 years after diagnosis of BC was praised as “*life saving*” by the mainstream media when the landmark ATLAS study [3] demonstrated the cumulative risk of dying of BC between 5 and 14 years after first diagnosis was 12.2% in the extended treatment group *vs* 15% in the standard treatment group, a reduction in absolute risk of 2.8% (or a reduction in BC mortality of 28 per 1,000 women). If risk of breast (and other) cancer is significantly increased in the protracted period when hyperinsulinemia is present before diabetes diagnosis, as well as the strong association between hyperinsulinemia and the metabolic syndrome, countless lives might be saved because more than 25% of the world's adult population would benefit from metformin's ability to reduce the insulin-related risk of cancer and cancer-related mortality. However, while cancer researchers and patients are eagerly awaiting the results of the NCIC Clinical Trials Group MA.32 study-an ongoing adjuvant trial of 3,640 women with early stage BC examining the effect of metformin *vs* placebo on invasive cancer-free survival and other outcomes with completed surgery and (neo)adjuvant chemotherapy [4]-there is growing uncertainty regarding the best models to predict the practical metformin posology that might be required for clinical activity in nondiabetic subjects.

On the one hand, numerous translational concerns have been expressed over the growing list of *in vitro* studies that are offently claimed clinically irrelevant because millimolar concentrations of metformin are typically required to induce anticancer effects [5–8], whereas the doses used in the clinical management of type 2 diabetes are normally in the range of 8-30 μM. However, the so-called metformin dosing paradox might not be translationally significant when considering that a dynamic relationship exists between metformin efficacy and chronic energy excess [9–12]. Because glucose availability promotes cancer aggressiveness while reducing metformin efficacy, maintenance of glucose homeostasis through energy restriction, exercise, and other pharmacological modalities is expected to enhance metformin responsiveness and clinical outcomes in cancer patients [11]. Alternatively, synthetic lethality could be exploited to overcome glucose-driven resistance to metformin by combining metformin with anti-angiogenesis agents that promote severe glucose deprivation in certain areas of the tumor [12]. On the other hand, although an initial report from the MA.32 trial has shown that, irrespective of the initial degree of insulin resistance in eligible non-diabetic women, metformin significantly improves metabolic parameters such as insulin and glucose at 6 months [4], it is commonly argued that the magnitude of such changes is small and may not be sufficient to alleviate the potential mitogenic and anti-apoptotic effects of insulin on occult or known cancers in patients without metabolic dysregulation. Because the pharmacology of metformin in the cancer setting has been largely unexplored, a brace of studies recently published in *Cell Metabolism* has attempted to shed new light on the metformin pharmacokinetic (PK) in mice bearing tumors, and its implications for human therapy [13, 14].

The studies led by Dowling and Chandel recapitulate earlier findings from our laboratory and others showing that mice i.p. injected with metformin have considerably higher plasma levels of metformin than those receiving the drug dissolved in the drinking water [15, 16] (Table 1). To evaluate the missing PK properties of metformin in the context of cancer therapy while concurrently excluding a role for insulin in its anti-tumor action in murine cancer models, we used breast xenotumors formed by *PIK3CA H1047R*-mutated MCF10DCIS.com cells (Cufí et al., 2013), which proliferate *in vitro* irrespective of the presence or absence of insulin, and form tumors that are refractory to dietary restriction (DR) *in vivo* [17, 18]. In a first cohort, cell line-derived xenograft (CDX) mice had *ad libitum* access to drinking water with 1.25 mg/mL metformin, which provided a daily oral dose of ~250 mg/kg. In a second cohort, metformin was administered to CDX mice by i.p. injection (200 mg/kg, once daily). The average plasma levels of metformin were evaluated using HPLC coupled to electrospray ionization quadrupole time-of-flight mass spectrometry at the end of an 8-week treatment when animals were sacrificed to evaluate tumor volume, mitotic activity, and anatomopathological features. BALB/c nude mice receiving metformin in the drinking water yielded plasma concentrations in the 5 μM range, an identical range to that reported by Chandel et al. [14] in C57NL/6J mice and in J:Nu mice bearing the metformin-responsive HCT116 *p53^−/−^* colon cancer tumor (Table 1). When metformin was measured 2 h after the last i.p. dose, metformin peaked in the plasma >100 fold higher than that found with the oral dosing schedule. Dowling et al. [13] now similarly report that the average plasma level of metformin in NOD/SCID mice xenografted with metformin-unresponsive HCT116 colorectal cancer cells was drastically higher in the i.p.-treated group (Table 1). Because the cation metformin is expected to accumulate up to 500-fold in mitochondria by virtue of the membrane potential, the low micromolar concentrations of plasma metformin in murine cancer models treated with the standard oral dosing of diabetic patients might translate into an anti-tumor activity exclusively attributable to metformin's ability to inhibit oxidative phosphorylation [13]. In our hands, such diabeto-biguanide activity modestly affected the growth of insulin-independent breast xenotumors, reaching a maximum of 43% at 4 weeks after inoculation of tumor cells and decreasing to 30-35% reduction toward the end of the treatment period [16, 18]. The apparent correlation between longer exposure to i.p.-delivered metformin, higher levels of plasma metformin, and higher efficacy in murine cancer models might suggests a re-evaluation of metformin administration regimens to optimize drug plasma levels and delivery to the tumor, but with the potential of involving mechanisms of action not strictly related with blockade of oxidative phosphorylation [14]. In our hands, such onco-biguanide activity increased in a temporal manner, reaching a highly significant >80% inhibition of tumor growth [16, 18].

**Table 1:** Summary of metformin posology in murine cancer models

		Route of administration		Plasma concentration (mean, [range])	
Study/Year	Animal strain	Oral (Dose/time)	i.p. (Dose/time)	Oral	i.p.
Memmott et al. (2010)	A/J	1000 mg/kg/13 weeks	250 mg/kg/13 weeks	13 μM	31 μM
Menendez et al. (2014)	BALB/c nu/nu	250 mg/kg/8 weeks	200 mg/kg/8 weeks	5 μM [3–6]	674 μM [387-1161]
Chandel et al. (2016)	C57BL/6J	250 mg/kg/2 weeks	-	5 μM	-
Chandel et al. (2016)	J:Nu	250 mg/kg/2 weeks	-	[3.2-12.4 μM]	-
Chandel et al. (2016)	NMRI nu/nu	-	350 mg/kg/2 weeks	-	7.5 μM
Dowling et al. (2016)	NOD/SCID	-	125 mg/kg/0.5 hr	-	184 μM [61-288]
Dowling et al. (2016)	NOD/SCID	1000 mg/kg/16 days	125 mg/kg/15 days	34 μM [2-126]	145 μM [66-215]

We need to anticipate the challenges that developing metformin and other onco-biguanides as *bona fide* anti-cancer metabolic therapeutics will face in the coming years (Table 2):

- First, the recent discovery that *in vivo* environment strictly dictates the metabolic phenotype of tumors in patients and mouse models [19] together with the contradictory findings obtained in patient-derived xenograft (PDX) models treated with metformin [20, 21], strongly indicate that clinically-translatable models remain to be developed to accurately assess the true potential of metformin-based therapeutic modalities. The use of immunocompromised mice or the fact that the subcutaneous compartment where tumors are placed in CDX and PDX is not analogous to the microenvironment within organs might significantly impact the response to metformin. Patient-derived orthotopic xenografts (PDOX) and 3D cancer tissue bioreactors for long-term organotypic culture and drug delivery might better approximate a metformin-targetable *in vivo* tumor microenvironment *ex vivo*.
- Second, effective design, analysis, and interpretation of PK and pharmacodynamic (PD) studies would enable a better understanding of the mechanism of metformin action and identify PK properties for further improvement. A valuable route to implement the successful development of metformin as an anti-cancer drug might involve PK/PD modeling of metformin in type 2 diabetics enrolled in early research phases of other oncology discovery projects involving metformin-related molecular targets (e.g., inhibitors of mTOR, PI3K) [22–24]. Such an approach could help elucidate the relationship between PK and PD, help to understand the mechanism of metformin action, and identify PK properties for further development and optimal usage of metformin in a cancer setting.
- Third, a major challenge in metformin posology and/or administration to optimize drug plasma levels is how to ensure that high metformin concentrations will cause tumor regression with minimal normal tissue toxicity. Although it is commonly acknowledged that such approaches will inevitably suffer from narrow therapeutic windows that might cause side effects such as lactic acidosis, we must consider the evidence that a Cochrane review and an analysis of the UK-based General Practice Research Database revealed no cases of fatal lactic acidosis and crude incidence rates of only 3.3 cases per 100,000 person-years among metformin users [25, 26]. Although it could be argued that additional side effects might be seen at supra-diabetic metformin concentrations, there have been no attempts to evaluate metformin-based synthetic lethal interactions sparing normal cells while selectively killing cancer cells. Hypothesis-driven and screening-based (e.g., chemical, siRNA/shRNA, CRISPR libraries) synthetic lethality approaches [27] would identify second-site molecular targets that could synergize with metformin-related metabolic targets and improve metformin efficacy, and might even provide new clinically relevant biomarkers for patient selection. A hypothesis-driven synthetic lethality approach recently conducted in our laboratory revealed the extreme vulnerability of cells deficient in the BC early onset gene *BRCA1* to NAD^+^ depletion after concurrent metformin [28] and nicotimamide phosphoribosyltransferase (NAMPT) inhibition, suggesting that the evaluation of metformin/NAMPT small molecule inhibitor combinations (e.g., FK866) to treat *BRCA1*-related BC may be warranted (manuscript in preparation).
- Fourth, identification of predictive biomarkers of response to metformin and precise patient selection is essential to ensure the measurement of true response rates without bias through inclusion of patients who do not respond to metformin simply because they are not suitable candidates to benefit from it. In the vast majority of ongoing metformin-based clinical trials, patient selection based on a particular biomarker has not been carried out, which may have improved the commonly observed poor response rates. In addition, the dysregulation and integration of multiple metabolic pathways in most tumors might trigger redundant/compensatory networks that could impact the effectiveness of metformin. Metformin-diagnostic co-development programs should be implemented to identify “decisive” stratification factors that have the potential to be an important tool for clinicians in relation to: 1) the identification of patients who are most likely to benefit from metformin; 2) the identification of patients likely to be at increased risk of serious adverse reactions as a result of metformin treatment; and 3) monitoring response to metformin to achieve improved safety or effectiveness. Such companion diagnostic assays, which should have a high degree of analytical validity before they can be released for routine clinical usage, might incorporate multi-metabolite panels based on the identification of metformin-driven metabolomic/fluxomic “fingerprints” of specific cellular events related with efficacy and safety. The exploration of the exo-metabolome to monitor, in real-time, biomarker/surrogate endpoints of metformin efficacy in liquid biopsies including urine [29] might optimize and accelerate the design of metformin-based personalized cancer therapies.

**Table 2:** Biguanides and cancer: Challenges ahead

CHALLENGE	PROPOSAL
(1) Clinically translatable models	CDX^a^, PDX^b^ → PDOX^c^, 3-D tissue bioreactors
(2) Implementation of PK/PD strategies	Metformin PK/PD in type 2 diabetics being enrolled in early research phases of oncology drugs targeting metformin-related molecular targets
(3) Therapeutic window and patient selection	Identification of synthetically lethal interactions to improve metformin-induced selective killing of cancer cells while sparing normal cells Using second-site molecular targets that sensitize cancer cells to metformin as biomarkers for patient selection
(4) Companion diagnostics	Identification of metabolomic/fluxomic signatures that can be used both to predict efficacy and safety (outcome) and to monitor the response to metformin

a CDX: Cell line-derived xenografts;

b PDX: Patient-derived xenografts;

c PDOX: Patient-derived orthotopic xenografts

Inadvertently perhaps, among the millions of type 2 diabetics with occult or known cancers and who have been prescribed metformin since the 1950s, thousands may have benefited from the anticancer properties of this first-line pharmacotherapy. *Quo vadis?* Now, researchers aim to move metformin from a “non-targeted” stage of cancer therapy that has been mostly developed retrospectively and empirically into a “targeted” therapy by following a biological rationale and a predefined mechanism of action. But, who might benefit from metformin? *Cui bono?* If metformin is on the leading edge of a new generation of metabolic targeted therapies, perhaps it is the right time to consider that the challenges facing the incorporation of metformin into the therapeutic armamentarium against cancer might be as complicated as targeting genetic aberrations, if not more so.

## References

[1] Goodwin PJ, Ennis M, Pritchard KI, Trudeau ME, Koo J, Madarnas Y, Hartwick W, Hoffman B, Hood N (2002). Fasting insulin and outcome in early-stage breast cancer: results of a prospective cohort study. J Clin Oncol.

[2] Goodwin PJ, Pritchard KI, Ennis M, Clemons M, Graham M, Fantus IG (2008). Insulin-lowering effects of metformin in women with early breast cancer. Clin Breast Cancer.

[3] Davies C, Pan H, Godwin J, Gray R, Arriagada R, Raina V, Abraham M, Medeiros Alencar VH, Badran A, Bonfill X, Bradbury J, Clarke M, Collins R (2013). Long-term effects of continuing adjuvant tamoxifen to 10 years versus stopping at 5 years after diagnosis of oestrogen receptor-positive breast cancer: ATLAS, a randomised trial. Lancet.

[4] Goodwin PJ, Parulekar WR, Gelmon KA, Shepherd LE, Ligibel JA, Hershman DL, Rastogi P, Mayer IA, Hobday TJ, Lemieux J, Thompson AM, Pritchard KI, Whelan TJ, Mukherjee SD, Chalchal HI, Oja CD, Tonkin KS, Bernstein V, Chen BE, Stambolic V (2015). Effect of metformin vs placebo on and metabolic factors in NCIC CTG MA.32. J Natl Cancer Inst.

[5] Vujic I, Sanlorenzo M, Posch C, Esteve-Puig R, Yen AJ, Kwong A, Tsumura A, Murphy R, Rappersberger K, Ortiz-Urda S (2015). Metformin and trametinib have synergistic effects on cell viability and tumor growth in NRAS mutant cancer. Oncotarget.

[6] Cuyàs E, Fernández-Arroyo S, Corominas-Faja B, Rodríguez-Gallego E, Bosch-Barrera J, Martin-Castillo B, De Llorens R, Joven J, Menendez JA (2015). Oncometabolic mutation IDH1 R132H confers a metformin-hypersensitive phenotype. Oncotarget.

[7] Loubière C, Goiran T, Laurent K, Djabari Z, Tanti JF, Bost F (2015). Metformin-induced energy deficiency leads to the inhibition of lipogenesis in prostate cancer cells. Oncotarget.

[8] Della Corte CM, Ciaramella V, Di Mauro C, Castellone MD, Papaccio F, Fasano M, Sasso FC, Martinelli E, Troiani T, De Vita F, Orditura M, Bianco R, Ciardiello F, Morgillo F (2016). Metformin increases antitumor activity of MEK inhibitors through GLI1 downregulation in LKB1 positive human NSCLC cancer cells. Oncotarget.

[9] Anisimov VN (2015). Metformin for cancer and aging prevention: is it a time to make the long story short?. Oncotarget.

[10] Chae YK, Arya A, Malecek MK, Shin DS, Carneiro B, Chandra S, Kaplan J, Kalyan A, Altman JK, Platanias L, Giles F (2016). Repurposing metformin for cancer treatment: current clinical studies. Oncotarget.

[11] Wahdan-Alaswad R, Fan Z, Edgerton SM, Liu B, Deng XS, Arnadottir SS, Richer JK, Anderson SM, Thor AD (2013). Glucose promotes breast cancer aggression and reduces metformin efficacy. Cell Cycle.

[12] Menendez JA, Oliveras-Ferraros C, Cufí S, Corominas-Faja B, Joven J, Martin-Castillo B, Vazquez-Martin A (2012). Metformin is synthetically lethal with glucose withdrawal in cancer cells. Cell Cycle.

[13] Dowling RJ, Lam S, Bassi C, Mouaaz S, Aman A, Kiyota T, Al-Awar R, Goodwin PJ, Stambolic V (2016). Metformin Pharmacokinetics in Mouse Tumors: Implications for Human Therapy. Cell Metab.

[14] Chandel NS, Avizonis D, Reczek CR, Weinberg SE, Menz S, Neuhaus R, Christian S, Haegebarth A, Algire C, Pollak M (2016). Are Metformin Doses Used in Murine Cancer Models Clinically Relevant?. Cell Metab.

[15] Memmott RM, Mercado JR, Maier CR, Kawabata S, Fox SD, Dennis PA (2010). Metformin prevents tobacco carcinogen--induced lung tumorigenesis. Cancer Prev Res (Phila).

[16] Menendez JA, Quirantes-Piné R, Rodríguez-Gallego E, Cufí S, Corominas-Faja B, Cuyàs E, Bosch-Barrera J, Martin-Castillo B, Segura-Carretero A, Joven J (2014). Oncobiguanides: Paracelsus’ law and nonconventional routes for administering diabetobiguanides for cancer treatment. Oncotarget.

[17] Kalaany NY, Sabatini DM (2009). Tumours with PI3K activation are resistant to dietary restriction. Nature.

[18] Cufí S, Corominas-Faja B, Lopez-Bonet E, Bonavia R, Pernas S, López IÁ, Dorca J, Martínez S, López NB, Fernández SD, Cuyàs E, Visa J, Rodríguez-Gallego E (2013). Dietary restriction-resistant human tumors harboring the PIK3CA-activating mutation H1047R are sensitive to metformin. Oncotarget.

[19] Davidson SM, Papagiannakopoulos T, Olenchock BA, Heyman JE, Keibler MA, Luengo A, Bauer MR, Jha AK, O'Brien JP, Pierce KA, Gui DY, Sullivan LB, Wasylenko TM (2016). Environment Impacts the Metabolic Dependencies of Ras-Driven Non-Small Cell Lung Cancer. Cell Metab.

[20] Lonardo E, Cioffi M, Sancho P, Sanchez-Ripoll Y, Trabulo SM, Dorado J, Balic A, Hidalgo M, Heeschen C (2013). Metformin targets the metabolic achilles heel of human pancreatic cancer stem cells. PLoS One.

[21] Lipner MB, Marayati R, Deng Y, Wang X, Raftery L, sO'Neil BH, Yeh JJ (2016). Metformin Treatment Does Not Inhibit Growth of Pancreatic Cancer Patient-Derived Xenografts. PLoS One.

[22] Davis NM, Sokolosky M, Stadelman K, Abrams SL, Libra M, Candido S, Nicoletti F, Polesel J, Maestro R, D'Assoro A, Drobot L, Rakus D, Gizak A (2014). Deregulation of the EGFR/PI3K/PTEN/Akt/mTORC1 pathway in breast cancer: possibilities for therapeutic intervention. Oncotarget.

[23] Yu G, Fang W, Xia T, Chen Y, Gao Y, Jiao X, Huang S, Wang J, Li Z, Xie K (2015). Metformin potentiates rapamycin and cisplatin in gastric cancer in mice. Oncotarget.

[24] Lau YK, Du X, Rayannavar V, Hopkins B, Shaw J, Bessler E, Thomas T, Pires MM, Keniry M, Parsons RE, Cremers S, Szabolcs M, Maurer MA (2014). Metformin and erlotinib synergize to inhibit basal breast cancer. Oncotarget.

[25] Salpeter SR, Greyber E, Pasternak GA, Salpeter Posthumous EE (2010). Risk of fatal and nonfatal lactic acidosis with metformin use in type 2 diabetes mellitus. Cochrane Database Syst Rev.

[26] Bodmer M, Meier C, Krähenbühl S, Jick SS, Meier CR (2008). Metformin, sulfonylureas, or other antidiabetes drugs and the risk of lactic acidosis or hypoglycemia: a nested case-control analysis. Diabetes Care.

[27] Thompson JM, Nguyen QH, Singh M, Razorenova OV (2015). Approaches to identifying synthetic lethal interactions in cancer. Yale J Biol Med.

[28] Cuyàs E, Fernández-Arroyo S, Alarcón T, Lupu R, Joven J, Menendez JA (2016). Germline BRCA1 mutation reprograms breast epithelial cell metabolism towards mitochondrial-dependent biosynthesis: evidence for metformin-based “starvation” strategies in BRCA1 carriers. Oncotarget.

[29] Liu Z, Yokoyama NN, Blair CA, Li X, Avizonis D, Wu XR, Uchio E, Youssef R, McClelland M, Pollak M, Zi X (2016). High Sensitivity of an Ha-RAS Transgenic Model of Superficial Bladder Cancer to Metformin Is Associated with ~240-Fold Higher Drug Concentration in Urine than Serum. Mol Cancer Ther.

